# Postoperative Bone Marrow Lesions (BMLs) Are Associated with Pain Severity in Patients Undergoing Open Wedge High Tibial Osteotomy (OWHTO)

**DOI:** 10.1155/2021/9938037

**Published:** 2021-07-07

**Authors:** Bo Zhu, Tong-Fu Wang, De-Sheng Chen, Jia-Wang Zhu, Zeng-Liang Wang, Jian-Gang Cao, Jun-Wei Zhao

**Affiliations:** Department of Sports Medicine and Arthroscopy, Tianjin Hospital of Tianjin University, Tianjin, China

## Abstract

The purpose of the study was to investigate the relationship between postoperative bone marrow lesions (BMLs) and pain severity in patients undergoing open wedge high tibial osteotomy (OWHTO). We reviewed the patients undergoing OWHTO between April 2018 and April 2020. The demographic and clinical data of patients were collected. Clinically, VAS and Knee injury and Osteoarthritis Outcome Score (KOOS) were used to assess pain level and functional outcomes of patients. The MRI Osteoarthritis Knee Score (MOAKS) was used to assess the total BMLs size in medial tibiofemoral (MTF), lateral tibiofemoral (LTF), and patellofemoral (PF) joints. 98 patients were enrolled in the study, including 57 male and 41 female patients. The VAS scores improved significantly from 6.1 ± 0.8 to 1.5 ± 0.9 (*p* < 0.001), and all subscales of KOOS improved significantly after surgery (*p* < 0.001). There were no significant differences between the pre- and postoperative total BML size of PF and LTF joints (*p* > 0.05). We observed significant improvements in the total BML size of MTF joint (*p* < 0.001). The VAS scores and KOOS pain scores improved better in patients without postoperative MTF joint BMLs (*p* < 0.001). Postoperative MTF joint BMLs were correlated with postoperative VAS (*p* < 0.001) and KOOS pain (*p* < 0.001). Our study demonstrates that MTF joint BMLs improved significantly after OWTHO. We confirmed that the presence of postoperative MTF joint BMLs are strongly associated with pain severity. The greater the improvement in postoperative MTF joint BMLs, the less pain. Our findings provide valuable understandings of OWHTO in the treatment of knee osteoarthritis (KOA) and potential future directions for KOA treatment approaches.

## 1. Introduction

Knee osteoarthritis (KOA) is a common chronic disease that causes pain, stiffness, and functional disability [1–4], among which, pain is the major cause leading individuals to seek medical care [5–8]. Many studies have demonstrated that knee pain could be relieved by open wedge high tibial osteotomy (OWHTO) in case of malalignment [9–12].

Despite the clinical efficacy of OWHTO for pain relief has been demonstrated, the underlying mechanisms and corresponding histological changes remain unclear. Many studies have shown that bone marrow lesions (BMLs) are strongly associated with pain in KOA [5, 13–15]. In a study conducted by Felson et al., 401 patients with KOA were investigated, and 272 of 351 (77.5%) patients with painful knees had BMLs, comparing with 15 of 50 (30%) patients without painful knees had BMLs [13]. Moreover, a few studies have demonstrated that BMLs of the knee were significantly improved after OWHTO [16, 17]. These findings prompted further analysis of the relevance between BMLs and pain states in patients undergoing OWHTO.

So far, only a few studies have analyzed the BML changes and their effect on the prognosis for patients undergoing OWTHO [16–18]. Yang et al. reviewed 105 patients who underwent OWHTO, and they did not find any correlation between preoperative bone marrow edema (BME) severity and postoperative outcomes [19]. To our knowledge, no studies have investigated the relationship between postoperative BMLs and pain severity in patients undergoing OWHTO.

This study is aimed at evaluating the relationship between postoperative BMLs and pain severity in patients undergoing OWHTO.

## 2. Materials and Methods

### 2.1. Patient Selection

We retrospectively reviewed the patients undergoing OWHTO between April 2018 and April 2020. The study was approved by the institutional ethics committee, and all patients signed consent forms. The inclusion criteria were (1) symptomatic medial KOA, (2) first OWHTO on the affected side, and (3) complete clinical data. The exclusion criteria were (1) revision surgery and (2) simultaneous bilateral OWHTO.

### 2.2. Perioperative Management

**Figure 1:**
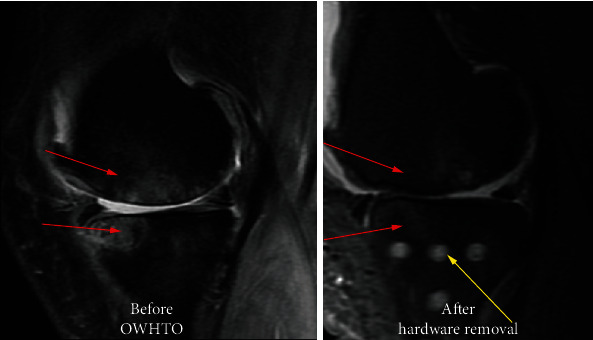
The BMLs in medial tibiofemoral joint improved significantly after surgery. The red arrow indicates the pre- and postoperative zone of BMLs, and the yellow arrow indicates the zone after hardware removal. BMLs: bone marrow lesions.

All patients received standard medical care, and all procedures were performed by the same surgical team. The osteotomy was performed under fluoroscopy, and the osteotomy site was fixed with the Tomofix plate system (Depuy *Synthes*, Eimattstrasse, Switzerland). Postoperative antibiotic prophylaxis (Intravenous cefuroxime, 1.5 g, *q8h for one day*) and analgesia were administered in all patients. For the prevention of venous thromboembolism, low molecule weight heparin (4250 IU, *qd*) was injected subcutaneously for 7 days, and rivaroxaban (10 mg *qd*) was administered during the subsequent 7 days. One year after hardware implantation, the hardware was removed, and magnetic resonance imaging (MRI) was performed (Figure 1).

### 2.3. MR Imaging and Analysis

**Figure 2:**
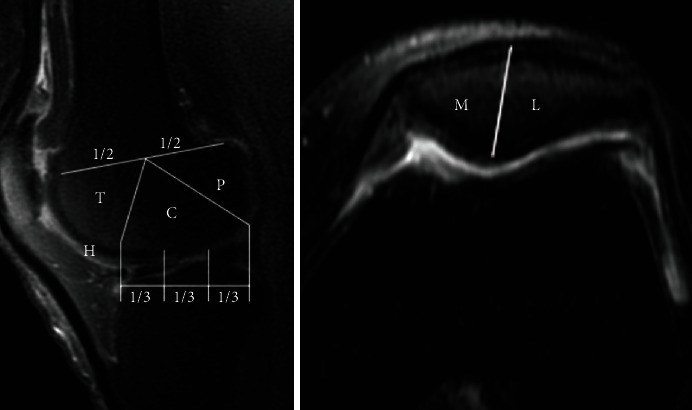
The LTF joint included five subregions: central (C) lateral femoral condyle, posterior (P) lateral femoral condyle, anterior subregion of tibia, central subregion of tibia, and posterior subregion of tibia. The maximum possible score in LTF joint was 15 and similarly for the MTF joint. The PF joint included four subregions: medial (M) portion of the patella, lateral (L) portion of the patella, medial trochlea (T) portion of femur, and lateral trochlea (T) portion of femur. The maximum possible BML size in PF joint was 12. LTF joint = lateral tibiofemoral joint, MTF joint = medial tibiofemoral joint, PF joint = patellofemoral joint.

MR images were analyzed by two expert musculoskeletal radiologists who were blind to any clinical information. The intraclass correlation coefficient (ICC) was calculated to assess interobserver variability. The MRI Osteoarthritis Knee Score (MOAKS) was used to assess the total BML size in medial tibiofemoral (MTF), lateral tibiofemoral (LTF), and patellofemoral (PF) joints. The criteria of the score are as follows: 0 = no BMLs in subregion; 1 = size of BMLs/subregional volume < 33%; 2 = size of BMLs/subregional volume < 66; 3 = size of BMLs/subregional volume > 66%. The maximum possible BMLs size was 15 in the MTF joint, 15 in the LTF joint, and 12 in the PF joint (Figure 2).

### 2.4. Data Collection

The demographic and clinical data of patients were collected, including gender, age, height, weight, body mass index (BMI), smoking, degree of correction, and size of osteotomy gap. Clinically, VAS and KOOS were used to assess pain level and functional outcomes of patients. MOAKS was used to assess the total BML size in MTF, LTF, and PF joints.

### 2.5. Statistical Analysis

The measurement data were expressed as mean value ± standard deviation (SD); the pre- and postoperative parameters were compared using paired *t*-tests and Wilcoxon test. An independent sample *t*-test was used to compare pain scores in patients with or without BMLs. The Pearson correlation test was used for correlation analysis. The SPSS 22.0 software was used for statistical analysis, and *p* < 0.05 was considered statistically significant.

## 3. Results

### 3.1. General Results

98 patients were enrolled in the study, including 57 male and 41 female patients. The mean age of the patients was 65.1 ± 5.7 years, and the mean BMI was 28.1 ± 3.4. The characteristics of the patients are shown in Table 1.

### 3.2. Results of the VAS, KOOS, and MOAKS

**Figure 3:**
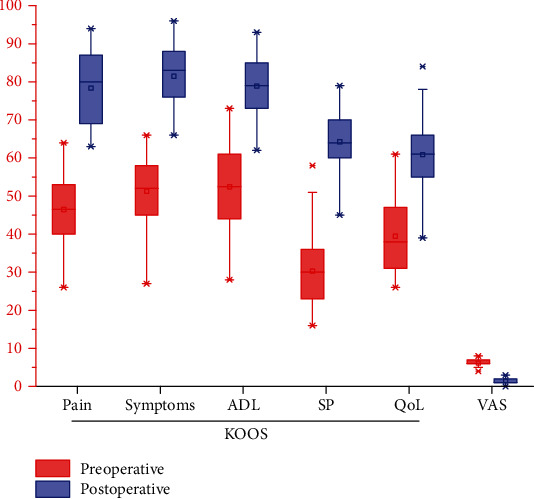
VAS scores and all subscales of KOOS improved significantly after surgery. KOOS: Knee injury and Osteoarthritis Outcome Score; ADL: activities of daily living; SR: sport and recreation; QoL: quality of life.

As shown in Table 2, the VAS scores improved significantly from 6.1 ± 0.8 to 1.5 ± 0.9 (*p* < 0.001), and all subscales of KOOS improved significantly after surgery (Figure 3). There were significant differences between smoker and nonsmoker regarding preoperative VAS scores (*p* = 0.040). There were no significant differences between smoker and nonsmoker regarding postoperative VAS scores and KOOS pain scores (*p* > 0.05).

**Figure 4:**
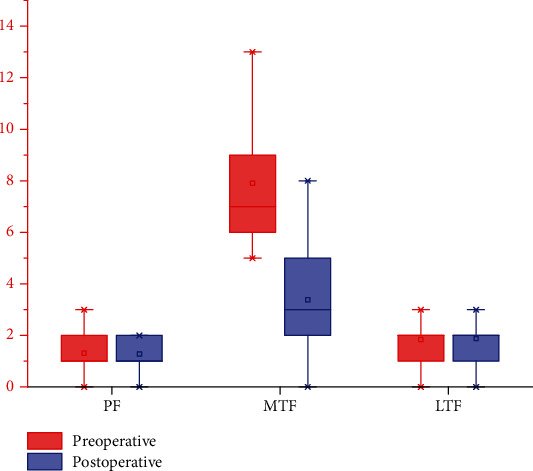
The total BML size in MTF joint improved significantly after surgery. BMLs: bone marrow lesions; PF joint: patellofemoral joint; MTF joint: medial tibiofemoral joint; LTF joint: lateral tibiofemoral joint.

ICC for all parameters were more than 0.8 in MRI measurements. As shown in Table 3, there was no significant difference between the pre- and postoperative total BML size in PF and LTF joints. We observed significant improvements in the total BML size in MTF joint (Figure 4). There were no significant differences between smoker and nonsmoker regarding the total BML size in PF joint, LTF joint, and MTF joint (*p* > 0.05).

### 3.3. The Relationship between Postoperative BMLs and Pain Severity

**Figure 5:**
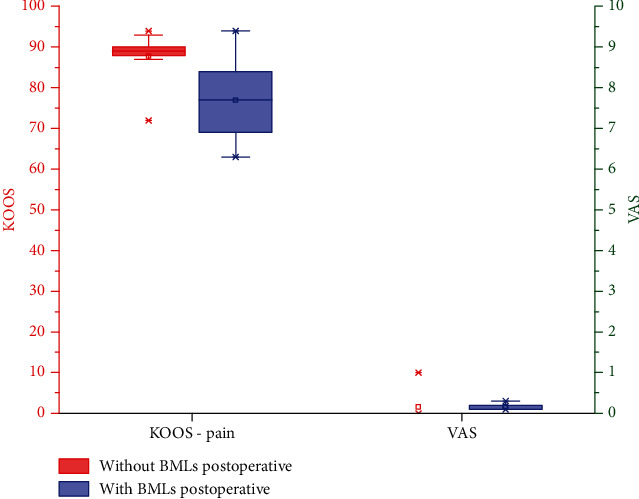
The VAS scores and KOOS pain scores improved better in patients without postoperative MTF joint BMLs. KOOS: Knee injury and Osteoarthritis Outcome Score; BMLs: bone marrow lesions.

All patients had preoperative MTF joint BMLs; in contrast, 13 patients had no postoperative MTF joint BMLs. The VAS scores were 1.7 ± 0.8 in patients with postoperative MTF joint BMLs and 0.2 ± 0.4 in patients without postoperative MTF joint BMLs. The KOOS pain scores were 76.9 ± 9.0 in patients with postoperative MTF joint BMLs and 87.8 ± 5.4 in patients without postoperative MTF joint BMLs. The independent sample *t*-test showed that the VAS scores and KOOS pain scores improved better in patients without postoperative MTF joint BMLs (Figure 5).

Pearson correlations showed that postoperative MTF joint BMLs are correlated with postoperative VAS (*r* = 0.945, *p* < 0.001) and postoperative KOOS pain (*r* = −0.472, *p* < 0.001).

## 4. Discussion

KOA is a degenerative joint disease, which causes pain and decreased physical function [1–4]. Knee pain is the major cause leading individuals to seek medical care [5–8]. The goal of KOA management is to relieve pain, improve knee function, and change the disease process. Although there are no approved drugs for changing KOA process, many interventions are available to address pain and function [1, 20, 21].

Many studies have demonstrated that knee pain could be relieved by OWHTO [9–12]. A systematic review found that clinical scores improved significantly after open or closed wedge high tibial osteotomy, including VAS, the American Knee Society Score, Hospital for Special Surgery Knee Score, and Lysholm score [22]. Identification of the mechanism of pain relief by OWHTO is important, an understanding of the mechanism may be helpful for targeted anti-KOA therapy and individualized therapy.

Some studies have shown that BMLs were more commonly observed in painful knees with OA than nonpainful knees [13, 14]. Meanwhile, increased BMLs were strongly associated with new onset frequent knee pain in nonpainful knees [23]. Zhang et al. investigated 651 painful knees and demonstrated that improved BMLs were strongly associated with pain relief in KOA [24]. These and other studies have implied that BMLs are the result of mechanical overload and thus inducing pain in KOA [5, 25–27].

The primary purpose of OWHTO was to reduce the mechanical load of medial compartment of the knee. Interestingly, a recent study implied that reducing mechanical load can decrease BMLs and relieve knee pain [28]. Our study demonstrates that MTF joint BMLs improved significantly after OWTHO, the greater the improvement in postoperative MTF joint BMLs, the less pain.

This study has several limitations: first, it is a small retrospective study. Second, the research is a single-center study; a further larger, multicenter research was needed to confirm our findings.

## 5. Conclusions

We confirmed that the presence of postoperative MTF joint BMLs are strongly associated with pain severity. The greater the improvement in postoperative MTF joint BMLs, the less pain. Our findings provide valuable information of OWHTO in the treatment of knee osteoarthritis (KOA) and potential future directions for KOA treatment approaches.

## Figures and Tables

**Table 1:** Patient characteristics.

Characteristics (*n* = 98)	
Gender (male/female)	57/41
Mean age (years)	65.1 ± 5.7
BMI (kg/m^2^)	28.1 ± 3.4
Smoking (%)	26 (26.5)
Alcohol consumption (%)	31 (31.6)
Hypertension (%)	45 (45.9)
Diabetes mellitus (%)	19 (19.4)
Degree of correction (°)	10.5 ± 1.6
Osteotomy gap (mm)	11.3 ± 2.8

BMI: body mass index.

**Table 2:** Preoperative and postoperative VAS and KOOS scores.

	Preoperative	Postoperative	*p* value
VAS	6.1 ± 0.8	1.5 ± 0.9	<0.001
KOOS pain	46.5 ± 8.2	78.3 ± 9.4	<0.001
KOOS symptoms	51.2 ± 9.1	81.5 ± 7.8	<0.001
KOOS ADL	52.4 ± 10.9	78.8 ± 8.2	<0.001
KOOS SR	30.3 ± 9.1	64.2 ± 7.2	<0.001
KOOS QoL	39.4 ± 9.2	60.9 ± 8.4	<0.001

KOOS: Knee injury and Osteoarthritis Outcome Score; ADL: activities of daily living; SR: sport and recreation; QoL: quality of life.

**Table 3:** Preoperative and postoperative MOAKS.

	Preoperative	Postoperative	*p* value
PF joint	1.3 ± 0.8	1.3 ± 0.7	0.566
LTF joint	1.8 ± 0.8	1.9 ± 0.7	0.083
MTF joint	7.9 ± 2.2	3.4 ± 2.0	<0.001

MOAKS: MRI Osteoarthritis Knee Score; PF joint: patellofemoral joint; MTF joint: medial tibiofemoral joint; LTF joint: lateral tibiofemoral joint.

## Data Availability

The datasets generated and analyzed during the present study are available from the corresponding author on reasonable request.
